# Supplementary material for: Sensitive detection of a bacterial pathogen using allosteric probe-initiated catalysis and CRISPR-Cas13a amplification reaction
Source: Nat Commun. 2020 Jan 14;11:267. doi: 10.1038/s41467-019-14135-9 (PMC6959245; doi:10.1038/s41467-019-14135-9)
Supplement: Supplementary file 1 — Supplementary Information [file 41467_2019_14135_MOESM1_ESM.pdf]

Supplementary information for "Sensitive detection of a bacterial pathogen using allosteric probe-initiated catalysis and CRISPR-Cas13a amplification reaction"

Jinjin Shen[1,2], Xiaoming Zhou[3], Yuanyue Shan[1,2], Huahua Yue[1,2], Ru Huang[1,2], Jiaming Hu[1,2*] and Da Xing[1,2*]

[1]MOE Key Laboratory of Laser Life Science & Institute of Laser Life Science, South China Normal University, Guangzhou 510631, China.

[2]College of Biophotonics, South China Normal University, Guangzhou 510631, China.

[3]School of Life Sciences, South China Normal University, Guangzhou 510631, China.

To whom correspondence should be addressed: jmhu@m.scnu.edu.cn (J.H.), xingda@scnu.edu.cn (D.X.)

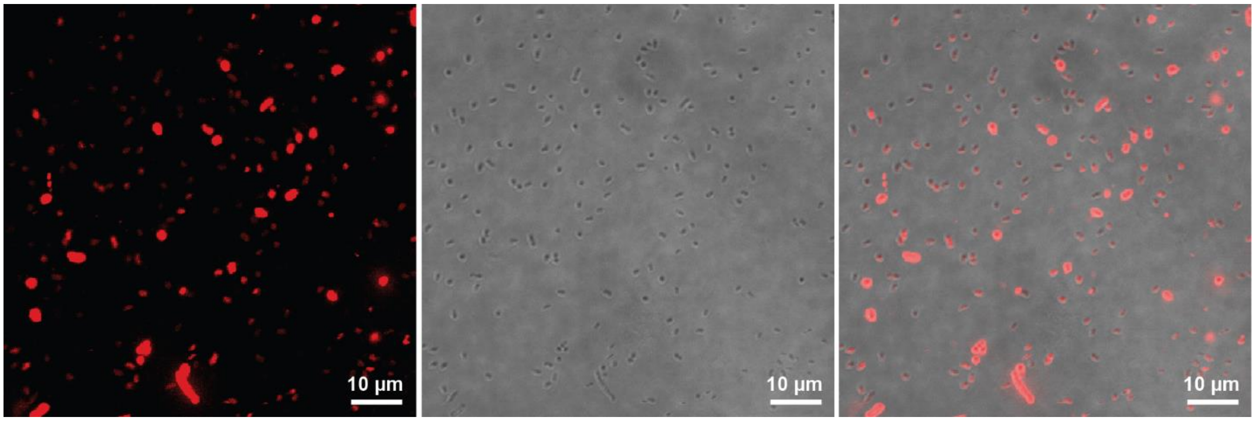

Supplementary Fig.1 | Laser scanning confocal microscope (LSCM) images of dual-labeled AP binding to *S.* Enteritidis.

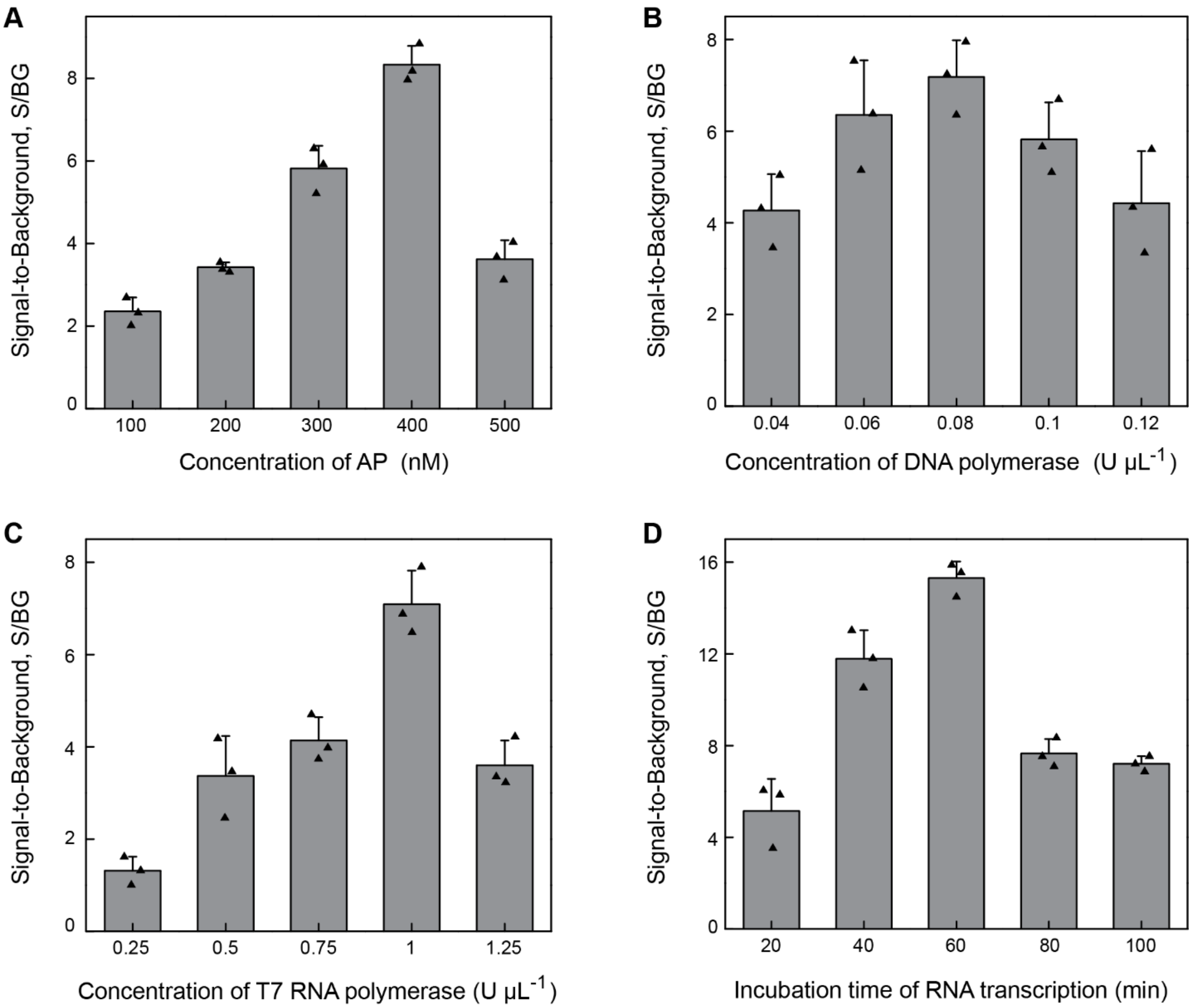

Supplementary Fig. 2 | Optimization of the reaction conditions for allosteric probe-initiated catalysis and CRISPR-Cas13a (APC-Cas) system for pathogen detection. (A) The concentration of allosteric probe (AP) tested in APC-Cas. (B) The concentration of Klenow Fragment DNA polymerase in extension reaction. (C) The concentration of T7 RNA polymerase in transcription amplification. (D) The incubation time of RNA transcription. Data represent mean ± s.d., n = 3, three technical replicates.

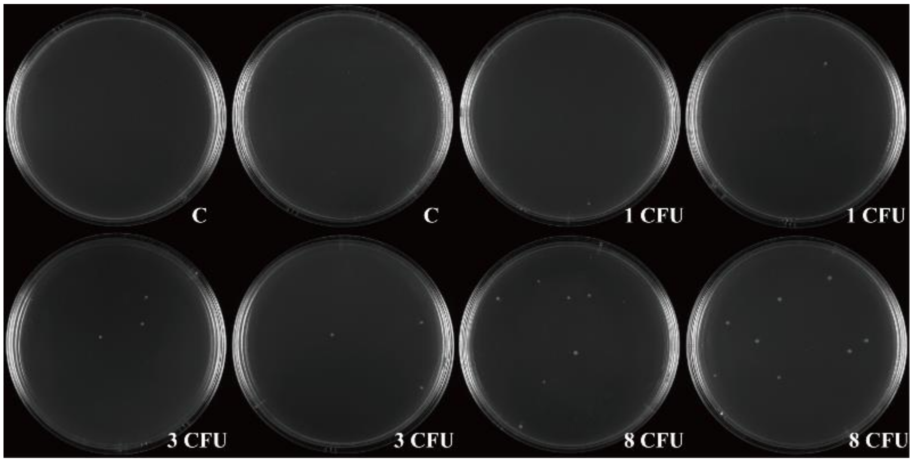

Supplementary Fig. 3 | The amount of *S.* Enteritidis were quantified by flat colony counting method.

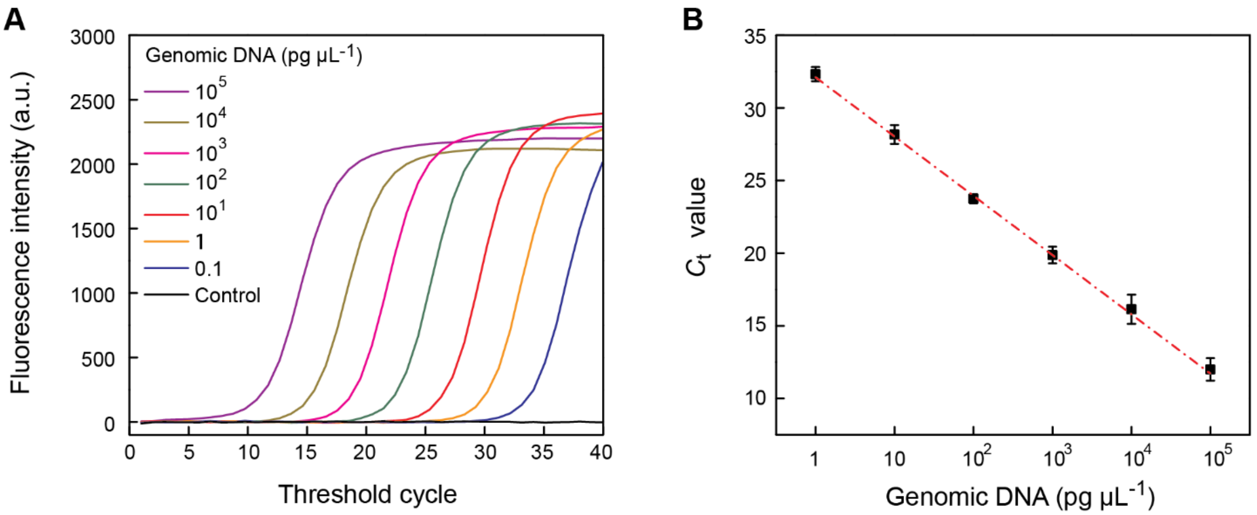

Supplementary Fig. 4 | Real-time PCR for detection of *S.* Enteritidis genomic DNA. (A) Real-time PCR was performed using the extracted *S.* Enteritidis genomic DNA in the range of 0.1 to 10^5 pg μL^{-1}. (B) Linear analysis of *S.* Enteritidis genomic DNA detection by real-time PCR. Data represent mean ± s.d., n = 3, three technical replicates.

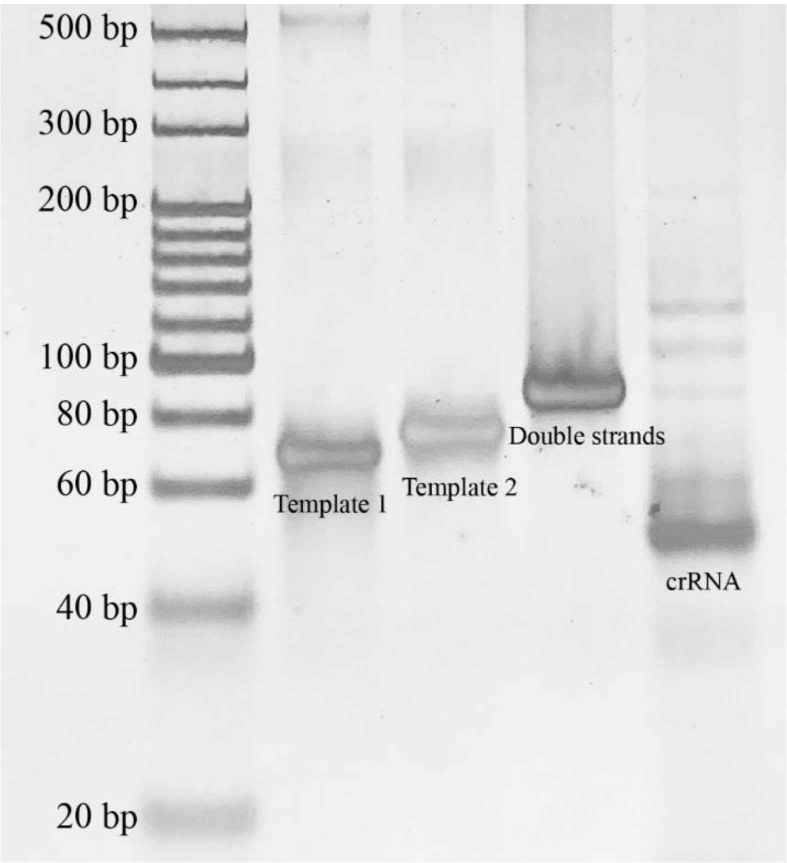

Supplementary Fig. 5 | The crRNA of CRISPR-Cas13a system was produced by *in vitro* transcription.

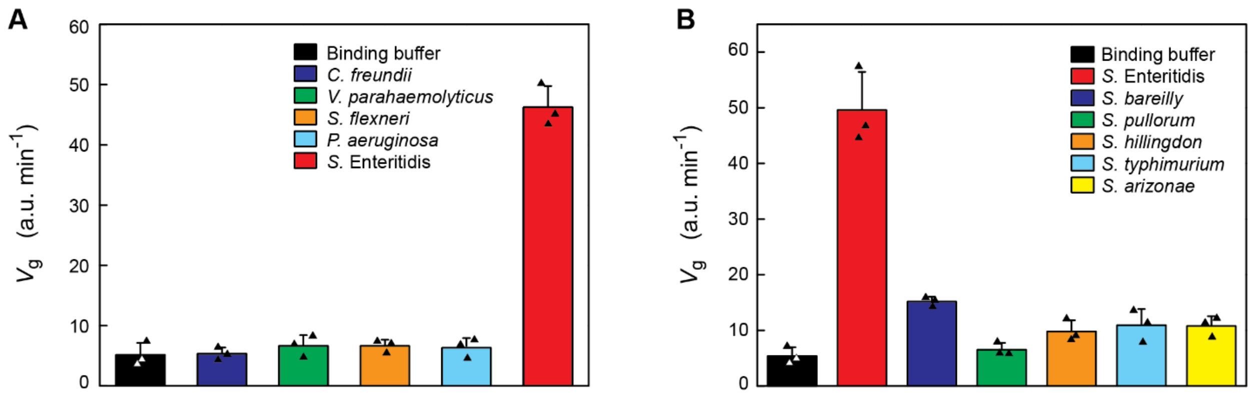

Supplementary Fig. 6 | Specificity test of APC-Cas system for *S.* Enteritidis. (A) Comparison of fluorescence growth rate (V_g) of APC-Cas system among *S.* Enteritidis and non-target gram-negative bacteria (*C. freundii*, *V. parahaemolyticus*, *S. flexneri*, *P. aeruginosa*). (B) Comparison of V_g of APC-Cas system among *S.* Enteritidis and other Salmonella enterica subspecies and serovars, including *S. bareilly*, *S. pullorum*, *S. hillingdon*, *S. typhimurium* and *S. arizonae*. Data represent mean ± s.d., n = 3, three technical replicates.

Strains	Serovars	Host species	Sources
CMCC 50040	*S.* Enteritidis	Human	Guangzhou Institute of Microbiology, China
CMCC 50041	*S.* Enteritidis	Human	National Center For Medical Culture Collections, China
CMCC 50335	*S.* Enteritidis	Mouse	National Center For Medical Culture Collections, China
CICC 21527	*S.* Enteritidis	Human	China Center of Industrial Culture Collection
CICC 24119	*S.* Enteritidis	Human	China Center of Industrial Culture Collection

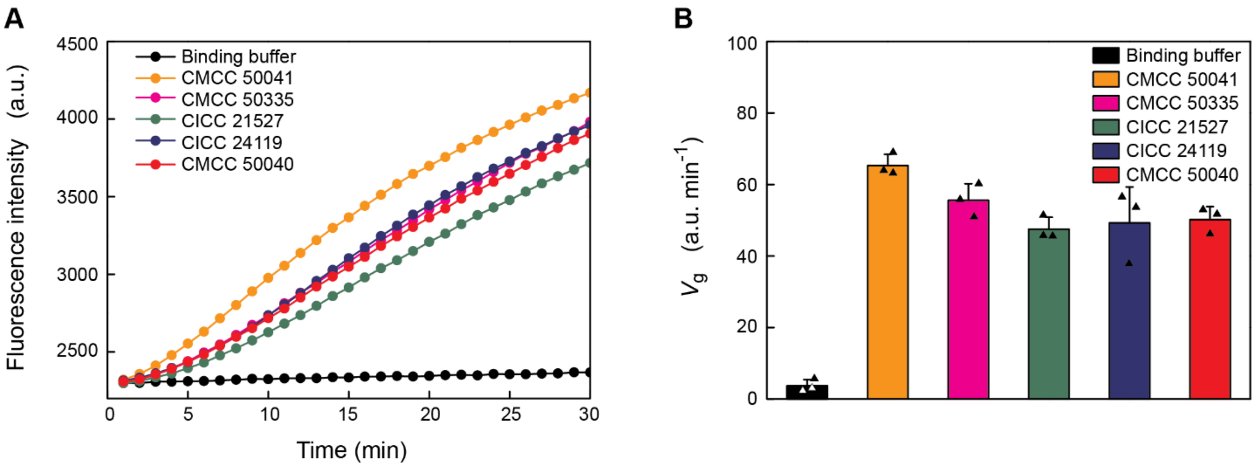

Supplementary Fig. 7 | Comparison of different *S.* Enteritidis strains within the same serotype using APC-Cas. (A) The fluorescence signal of APC-Cas for detection of five *S.* Enteritidis strains. (B) Fluorescence growth rate (V_g) of APC-Cas for detection of five *S.* Enteritidis strains. Data represent mean ± s.d., n = 3, three technical replicates.

Supplementary Table 1 | Single-stranded DNA and RNA sequences used in this study

Name	(abbreviation)	Nucleic acid sequence, listed 5' to 3'
Allosteric probe with 8 stem-length	(8-stem AP)	TCGGCAACAAGGTCACCCGGAGAAGATCGGTGGTC AAACTGCATAGGTAGTCCAGAAGCCCTATAGTGAGT CGTATTAAGGGCTTC-P
Allosteric probe with 9 stem-length	(9-stem AP)	TCGGCAACAAGGTCACCCGGAGAAGATCGGTGGTC AAACTGCATAGGTAGTCCAGAAGCCCTATAGTGAGT CGTATTAAGGGCTTCT-P
Allosteric probe with 10 stem-length	(10-stem AP)	TCGGCAACAAGGTCACCCGGAGAAGATCGGTGGTC AAACTGCATAGGTAGTCCAGAAGCCCTATAGTGAGT CGTATTAAGGGCTTCTG-P
Allosteric probe with 11 stem-length	(11-stem AP)	TCGGCAACAAGGTCACCCGGAGAAGATCGGTGGTC AAACTGCATAGGTAGTCCAGAAGCCCTATAGTGAGT CGTATTAAGGGCTTCTGG-P
Allosteric probe with 13 stem-length	(13-stem AP)	TCGGCAACAAGGTCACCCGGAGAAGATCGGTGGTC AAACTGCATAGGTAGTCCAGAAGCCCTATAGTGAGT CGTATTAAGGGCTTCTGGAC-P
Allosteric probe with 14 stem-length	(14-stem AP)	TCGGCAACAAGGTCACCCGGAGAAGATCGGTGGTC AAACTGCATAGGTAGTCCAGAAGCCCTATAGTGAGT CGTATTAAGGGCTTCTGGACT-P
Allosteric probe with 16 stem-length	(16-stem AP)	TCGGCAACAAGGTCACCCGGAGAAGATCGGTGGTC AAACTGCATAGGTAGTCCAGAAGCCCTATAGTGAGT CGTATTAAGGGCTTCTGGACTAC-P
Primer	(Primer)	GAAGCCCT
Template strand 1 of crRNA	(T1-crRNA)	GCCCTTAATACGACTCACTATAGGGGGACCACCCCA AAAATGAAGGGGACTAAAACTGGTCAAACTGCATAG GTAGTCCAGAAG
Template strand 2 of crRNA	(T2-crRNA)	CGGGAATTATGCTGAGTGATATCCCCCTGGTGGGGT TTTTACTTCCCCTGATTTTGACCAGTTTGACGTATCC ATCAGGTCTTC
crRNA	(crRNA)	GGACCACCCCAAAAAUGAAGGGGACUAAAACUGGU CAAACUGCAUAGGUAGUCCAGAAG
Reporter probe	(QF-RNA)	/FAM/UUUUU/BHQ1/
Dual-labeled AP	(QF-AP)	TCGGCAACAAGGTCACCCGGAGAAGATCGGTGGTC AAACTGCATAGGTA/BHQ2/GTCCAGAAGCCCTATAGT GAGTCGTATTAAGGGCTTCTGGAC/Cy5/
Primer'-forward (for sefA)	(sefA-P'-F)	GTGGTTCAGGCAGCGGTTAC
Primer'-reverse (for sefA)	(sefA-P'-R)	GCAAGCCCGTCAATTCCAGTAT

The DNA sequence marked in purple colour represents aptamer domain; the DNA sequence marked in yellow colour represents T7 promoter domain; the DNA sequence marked in blue colour represents primer binding site domain.

Supplementary Table 2 | Kinetic parameters of seven allosteric probe (AP) with varied stem length

Name	ΔG (kcal/mol)	Tm (°C)	ΔH (kcal/mol)	ΔS (cal/K·mol)
8-stem AP	-9.02	49.7	-118.1	-365.85
9-stem AP	-9.56	50.1	-123.3	-381.49
10-stem AP	-11.31	52	-136.4	-419.56
11-stem AP	-13.04	60.5	-122.7	-367.8
13-stem AP	-15.65	62.8	-139	-413.72
14-stem AP	-16.97	63.7	-147.8	-438.81
16-stem AP	-18.73	63.8	-162.6	-482.54

Supplementary Table 3 | Table associated with ROC curve analysis depicted in Fig. 4F.

Parameter	AUC	Cut-off value	Sensitivity	Specificity	95% CI
APC-Cas (V_g)	1	≥ 9.22	1	1	1.000 - 1.000
RT-PCR (C_t)	0.923	≤ 37.1	0.850	0.850	0.839 - 1.000

Supplementary Table 4 | Table associated with ROC curve analysis depicted in Fig. 5D.

Parameter	AUC	Cut-off value	Sensitivity	Specificity	95% CI
APC-Cas (V_g)	0.997	≥ 5.48	0.98	1	0.986 - 1.000

Supplementary Table 5 | APC-Cas cost analysis.

Reaction of APC-Cas	Component	Amount	Vendor	Cost ($)	Fraction used/reaction	Cost/1000 reactions ($)
Primary amplification	AP	2 OD	Sangon Biotech	67.63	4.54E-04	30.7162
	primer	2 OD	Sangon Biotech	7.05	7.50E-05	0.528375
	dNTP	250 µL	Takara	11.27	8.00E-04	9.0176
	KF	200 U	NEB	72.14	4.00E-03	288.5632
	RNase-free Water	500 mL	Sangon Biotech	7.50	1.00E-05	0.074959
Secondary amplification	rNTP	500 µL	NEB	10.93	1.00E-02	109.3384
	T7 RNA Polymerase	2500 U	NEB	314.35	4.00E-04	267.71
	Recombinant RNase Inhibitor	500 U	Takara	13.39	2.00E-02	125.7392
Tertiary amplification	Cas13a	20 nmol	custom purification	95.11	5.00E-06	0.475538
	RNase-free Water	500 mL	Sangon Biotech	7.50	1.00E-05	0.074959
	Reaction buffer	500 µL	Takara	2.11	1.00E-03	2.1135
	crRNA	100 µL	custom transcription	10.38	5.00E-05	0.519179
	Reporter probe	2 OD	Takara	276.16	8.40E-05	23.20623
					**Total cost/ 1000 reactions ($)**	**858.0773**
