# Supplementary material for: Sensitive detection of a bacterial pathogen using allosteric probe-initiated catalysis and CRISPR-Cas13a amplification reaction
Source: Nat Commun. 2020 Jan 14;11:267. doi: 10.1038/s41467-019-14135-9 (PMC6959245; doi:10.1038/s41467-019-14135-9)
Supplement: Supplementary file 3 — Reporting Summary [file 41467_2019_14135_MOESM3_ESM.pdf]

nature research

Corresponding author(s): Jiaming Hu

Last updated by author(s): Dec 6, 2019

Reporting Summary

Nature Research wishes to improve the reproducibility of the work that we publish. This form provides structure for consistency and transparency in reporting. For further information on Nature Research policies, see Authors & Referees and the Editorial Policy Checklist .

Statistics

For all statistical analyses, confirm that the following items are present in the figure legend, table legend, main text, or Methods section.

n/a	Confirmed	
☐	☒	The exact sample size (*n*) for each experimental group/condition, given as a discrete number and unit of measurement
☐	☒	A statement on whether measurements were taken from distinct samples or whether the same sample was measured repeatedly
☐	☒	The statistical test(s) used AND whether they are one- or two-sided<br>*Only common tests should be described solely by name; describe more complex techniques in the Methods section.*
☐	☒	A description of all covariates tested
☐	☒	A description of any assumptions or corrections, such as tests of normality and adjustment for multiple comparisons
☐	☒	A full description of the statistical parameters including central tendency (e.g. means) or other basic estimates (e.g. regression coefficient) AND variation (e.g. standard deviation) or associated estimates of uncertainty (e.g. confidence intervals)
☐	☒	For null hypothesis testing, the test statistic (e.g. *F*, *t*, *r*) with confidence intervals, effect sizes, degrees of freedom and *P* value noted<br>*Give P values as exact values whenever suitable.*
☒	☐	For Bayesian analysis, information on the choice of priors and Markov chain Monte Carlo settings
☐	☒	For hierarchical and complex designs, identification of the appropriate level for tests and full reporting of outcomes
☐	☒	Estimates of effect sizes (e.g. Cohen's *d*, Pearson's *r*), indicating how they were calculated

Our web collection on statistics for biologists contains articles on many of the points above.

Software and code

Policy information about availability of computer code

Data collection	*Provide a description of all commercial, open source and custom code used to collect the data in this study, specifying the version used OR state that no software was used.*
Data analysis	The GraphPad Prism version 5.0, IBM SPSS Statistics version 19.0 and Origin 8 were used to analyse the data in this study.

For manuscripts utilizing custom algorithms or software that are central to the research but not yet described in published literature, software must be made available to editors/reviewers. We strongly encourage code deposition in a community repository (e.g. GitHub). See the Nature Research guidelines for submitting code & software for further information.

Data

Policy information about availability of data

All manuscripts must include a data availability statement. This statement should provide the following information, where applicable:

- Accession codes, unique identifiers, or web links for publicly available datasets
- A list of figures that have associated raw data
- A description of any restrictions on data availability

The data are available within this manuscript and its Supplementary Information files, or from the corresponding author upon reasonable request. The final dataset is available from Figshare; DOI: 10.6084/m9.figshare.11309750

Field-specific reporting

Please select the one below that is the best fit for your research. If you are not sure, read the appropriate sections before making your selection.

Life sciences study design

All studies must disclose on these points even when the disclosure is negative.

Sample size	Sample size for each study was chosen based on literature documentation of similar well-characterized experiments, and no statistical method was used to predetermine sample size.
Data exclusions	No data was excluded from the analyses.
Replication	All attempts at replication were successful in this study.
Randomization	All samples in this study were randomly allocated into each experimental group.
Blinding	The investigators of this work were blinded to group allocation during data collection and analysis, as mentioned in the manuscript that the 40 milk samples used in this study were prepared by a third party as double-blind preparation.

Reporting for specific materials, systems and methods

We require information from authors about some types of materials, experimental systems and methods used in many studies. Here, indicate whether each material, system or method listed is relevant to your study. If you are not sure if a list item applies to your research, read the appropriate section before selecting a response.

Materials & experimental systems

n/a	Involved in the study	
☒	☐	Antibodies
☒	☐	Eukaryotic cell lines
☒	☐	Palaeontology
☐	☒	Animals and other organisms
☒	☐	Human research participants
☒	☐	Clinical data

Methods

n/a	Involved in the study	
☒	☐	ChIP-seq
☒	☐	Flow cytometry
☒	☐	MRI-based neuroimaging

Animals and other organisms

Policy information about studies involving animals; ARRIVE guidelines recommended for reporting animal research

Laboratory animals	Twelve 8- to 10-week-old C57BL/6 mice were selected randomly without gender orientation.
Wild animals	The study did not involve wild animals.
Field-collected samples	The study did not involve samples collected from the field.
Ethics oversight	All animal studies were conducted in accordance with the guidelines of the National Regulation of China for Care and Use of Laboratory Animals, and were performed with the approval of Scientific Research Ethics Committee of South China Normal University (South China Normal University, Guangzhou, China).

Note that full information on the approval of the study protocol must also be provided in the manuscript.
